# Secondary injury to distal regions after intracerebral hemorrhage influence neurological functional outcome

**DOI:** 10.18632/aging.102880

**Published:** 2020-03-08

**Authors:** Kangping Song, Xiaojie Liu, Qiuyue Zheng, Lingling Zhang, Hongying Zhang, Hailong Yu, Yan Zhu, Li-An Huang, Yingzhu Chen

**Affiliations:** 1 Department of Neurology, Institute of Clinical Neuroscience, The First Affiliated Hospital, Jinan University, Guangzhou 510632, Guangdong, China; 2 Department of Neurology, Clinical Medical College, Yangzhou University, Yangzhou 225000, Jiangsu, China; 3 Medical Imaging Center, Clinical Medical College, Yangzhou University, Yangzhou 225000, Jiangsu, China

**Keywords:** 1H-MRS, cognitive functional outcome, DTI, ICH, motor functional outcome

## Abstract

Although many studies have focused on functional impairment after intracerebral hemorrhage, little is known about the relationship between secondary injuries to distal regions and neurological function. Our study aimed to evaluate the secondary injuries after intracerebral hemorrhage and explore their relationship to neurological functional outcome. Twenty-one patients with hemorrhages in supratentorial, deep locations and 10 healthy subjects were recruited. Longitudinal examinations of diffusion tensor imaging, hydrogen proton magnetic resonance spectroscopy imaging and neuropsychological assessment were performed after weeks 1 and 12 to elucidate the relationship between magnetic resonance imaging parameters and neurologic outcomes. By week 12, motor function had significantly improved, but cognitive function had deteriorated compared to week 1. Fractional anisotropy values for the ipsilateral cerebral peduncle correlated with motor function at week 1. No significant correlation between fractional anisotropy for the ipsilateral cerebral peduncle and the Fugl-Meyer Motor Scale was found at week 12. Fractional anisotropy values for the ipsilateral hippocampus were related to the Montreal Cognitive Assessment and Mini-Mental State Examination at weeks 1 and 12. Deep supratentorial hemorrhage may result in injury to distal regions, which correlate with impaired motor and cognitive function.

## INTRODUCTION

More than 70% of the intracerebral hemorrhage (ICH) survivors live with movement dysfunction [1], and over 60% of these patients suffer cognitive impairment [2, 3]. Multimodal magnetic resonance imaging (MRI) plays a significant role in the diagnosis and treatment of central nervous system disease; by combining several kinds of MRI sequences, one can obtain information on both anatomical structure and metabolism.

Diffusion tensor imaging (DTI) is a noninvasive tool to detect the distribution and integrity of the white matter fiber [4]. Cho et al [5] found that motor function 6 months after ICH onset is related to the degree of nerve fiber damage. Other studies demonstrated the predictive value of DTI for motor function after ICH [6–8]. But there are still problems, including the controversial of optimal threshold of fractional anisotropy (FA) values for distinguishing clinical outcomes and the optimal times for examination [7, 8]. In addition, although DTI has been used in many studies to explore the mechanisms underlying cognitive impairment after ischemic stroke [9], the correlation between cognitive deficiency and secondary injuries after ICH has not attracted much attention.

Imaging using hydrogen proton magnetic resonance spectroscopy (1H-MRS) is a valuable tool for detecting metabolites such as N-acetylaspartate (NAA), creatine (Cr), myo-inositol (mI), lactic acid (Lac) and choline (Cho), among others. Grachev et al [10] found that patients who suffered a hemorrhage in the left temporal parietal junction area exhibited lower NAA and mI levels in the ipsilateral orbitofrontal cortex along with decreased cognition. Several other studies suggest that the NAA/Cr ratio is predictive of the risk of transition from mild cognitive impairment (MCI) to dementia [11, 12] and plays an important role in the identification of different types of MCI [13, 14]. While the published studies showed that 1H-MRS plays a significant role in early diagnosis and assessment of cognitive impairment, we wonder about the possible role of abnormal brain metabolism in a specific group of patients exhibiting cognitive decline after ICH.

The corticospinal tract is concentrated on the cerebral peduncle and plays a critical role in motor function. The hippocampus is a well-known area related to cognition, memory and spatial navigation [15], and it often suffers a secondary injury, as this area is extremely sensitive to ischemia [16, 17]. We therefore conducted this study to detect longitudinal changes in remote regions after local ICH, especially the cerebral peduncle and hippocampus, and used multimodal MRI to explore the relationship between these secondary injuries and neurological function.

## RESULTS

### Demographic data and neurological function

No significant differences in sex, age, education level or other risk factors were found between ICH and control groups (Table 1). Motor and cognitive function were intact in all control subjects. Obvious motor and cognitive deficits were found in patients 1 week and 12 weeks after ICH. However, ICH patients’ motor function during week 12 was significantly improved compared to week 1, whereas cognitive function had deteriorated by week 12 compared to week 1 (Table 2).

**Table 1:** Demographic data for the ICH and control groups.

**Variables**	**ICH (n = 21)**	**Control (n = 10)**	***P* value**
Age (years)	59.29 ± 9.89	58.00 ± 11.88	0.771
Male (n, %)	16 (76.19)	6 (60.00)	0.417
Smoking (n, %)	4 (19.05)	2 (20.00)	1.000
Drinking (n, %)	2 (9.52)	1 (10.00)	1.000
Hypertension (n, %)	17 (80.95)	5 (50.00)	0.105
Diabetes (n, %)	2 (9.52)	3 (30.00)	0.296
Education level (n, %)			0.511
Primary school	10 (47.62)	4 (40)	
Junior middle school	7 (33.33)	3 (30)	
senior middle school	4 (19.05)	2 (20)	
University	0	1 (10)	
Hematoma volume (ml)	7.02±4.70	/	

ICH : intracranial hemorrhage. * *P*<0.05.

**Table 2:** Neurologic function in the ICH and control groups at weeks 1 and 12.

	**ICH**		**Control**	***P*1**	***P*2**	***P*3**
	**Week 1**	**Week 12**				
NIHSS	5.9±4.19	1.62±0.81	0	0.000*	0.000*	0.000*
FMS	56.1±19.37	87.62±8.53	100	0.000*	0.000*	0.000*
MoCA	21.10±4.58	16.71±5.46	28.50±1.18	0.000*	0.000*	0.008*
MMSE	25.95±2.53	23.00±3.39	29.40±0.84	0.000*	0.000*	0.003*

*P*1 indicates ICH vs. control at 1^st^ week; *P*2 indicates ICH vs. control at 12^th^ week; *P3* indicates 12^th^ week vs. 1^st^ week among ICH patients; *P<0.05.

### DTI data analysis

**Figure 1:**
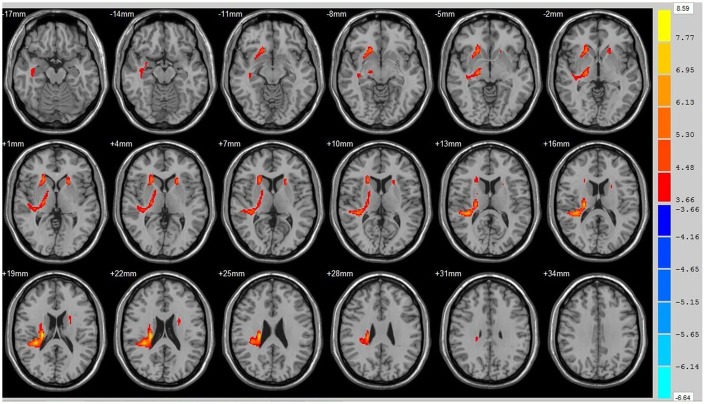
**Brain areas with decreased FA value at ICH patients at 1st w.** Orange means the brain areas which FA value decreased compared with control group at1st w, Threshold=3.6594.

**Figure 2:**
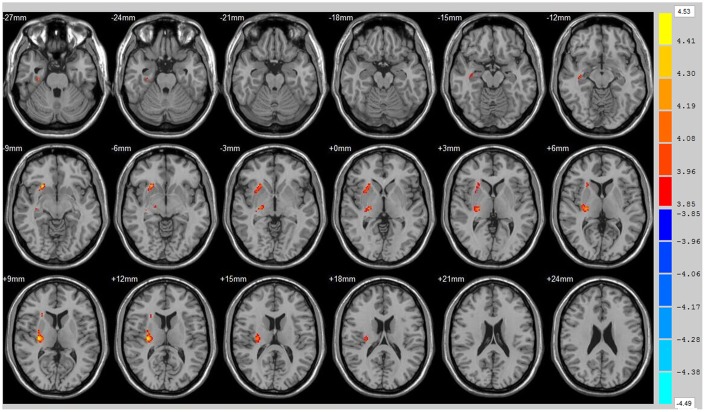
**Brain areas with decreased FA value of ICH patients at 12th w.** Orange means brain areas with decreased FA value of ICH patients compared with control group at 12th w, Threshold=3.8495.

**Figure 3:**
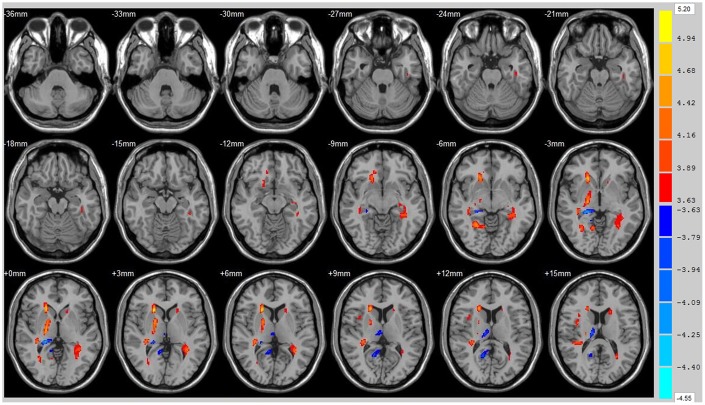
**Brain areas with decreased FA value of ICH patients at 1st w and 12th w.** Brain areas with increased FA value (Orange) and decreased FA value (Blue) of ICH patients at 12th w compared with 1st w, Threshold=3.6335.

Using the whole brain analysis, we found that 1 week after ICH, patients displayed lower FA values in two brain clusters that included the ipsilateral temporal lobe, frontal lobe, insula, lenticular nucleus, putamen, caudate nucleus, hippocampus and thalamus than did the controls (Figure 1 and Supplementary Table 1). Two clusters with decreased FA values were also observed at week 12 (Figure 2 and Supplementary Table 2). Moreover, 12 weeks after ICH, six clusters (ipsilateral frontal lobe, lenticular nucleus, caudate nucleus, putamen, globus pallidus, and the contralateral parietal lobe, temporal lobe and hippocampus) exhibited higher FA (Figure 3, orange and Supplementary Table 3) while two clusters (ipsilateral limbic lobe, posterior cingulate gyrus and hippocampus) exhibited lower FA than at week 1 (Figure 3, blue and Supplementary Table 4).

**Figure 4:**
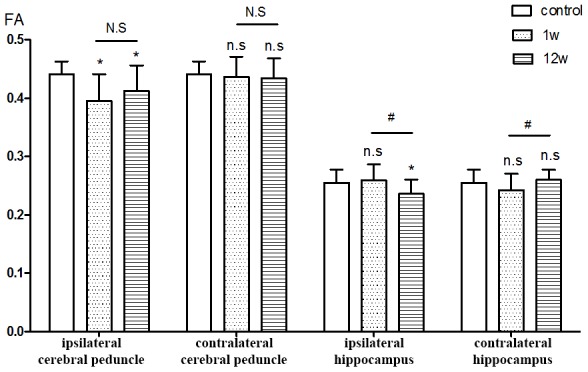
**ROIs analysis of FA value.** *P<0.05, compared with control group; n.s P>0.05 compared with control group; # P<0.05, compared with 1st w; N.S P>0.05 compared with 1st w.

**Figure 5:**
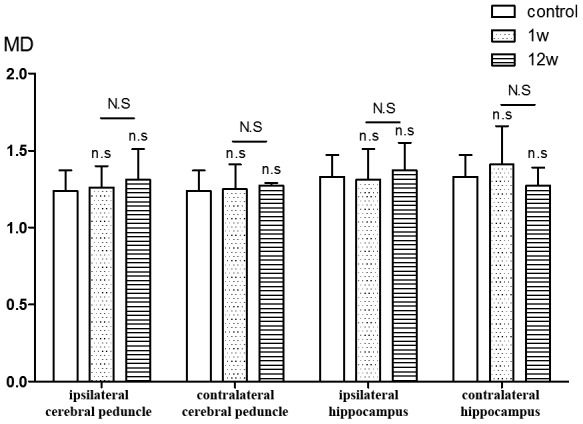
**ROIs analysis of MD value.** n.s P>0.05 compared with control group; N.S P>0.05 compared with 1st w.

Analysis of regions of interest (ROIs) revealed that as compared to control, FA values for the ipsilateral cerebral peduncle were reduced 1 week (*P*=0.008) and 12 weeks (*P*=0.020) after ICH. No significant differences from control were detected in the contralateral cerebral peduncle FA value at either time point. The ipsilateral hippocampus FA value at week 12 was obviously lower than in the control group (*P*=0.036), though no difference was detected at week 1. No significant difference was found between the contralateral hippocampus in the ICH and control groups at either time point. When analyzing the changes in FA values in ROIs in ICH patients over time, we found that the FA value for the ipsilateral hippocampus was significantly lower at week 12 than week 1 (*P*=0.004), while conversely the FA value for the contralateral hippocampus was significantly higher at week 12 than week 1 (*P*=0.020). The contralateral cerebral peduncle showed no obvious difference from control at either time point (Figure 4). In addition, no significant differences in the mean diffusivity (MD) value were detected between the ICH and control group at either week 1 or 12 (Figure 5).

### Association between FA values and neurological functional outcomes

Spearman analysis revealed that the patients’ ipsilateral cerebral peduncle FA value at week 1 correlated significantly with Fugl-Meyer Motor Scale (FMS) at both week 1 and 12 (r_1w_ = 0.494, *P*_1w_ = 0.023; r_12 w_ = 0.460, *P*_12 w_ = 0.036), whereas no correlation was detected between the ipsilateral cerebral peduncle FA value at week 12 and FMS (r_12w_ =0.391, *P*_12w_=0.201). At both week 1 and 12, the patients’ ipsilateral hippocampus FA value correlated significantly with the Montreal Cognitive Assessment (MoCA, r_1w_ = 0.569, *P*_1w_ = 0.007; r_12w_ = 0.644, *P*_12w_ = 0.002) and the Mini-Mental State Examination (MMSE, r_1w_ = 0.550, *P*_1w_ = 0.010; r_12w_ = 0.636, *P*_12w_ = 0.002) (Supplementary Figure 1).

### ^1^H-RMS analysis

**Figure 6:**
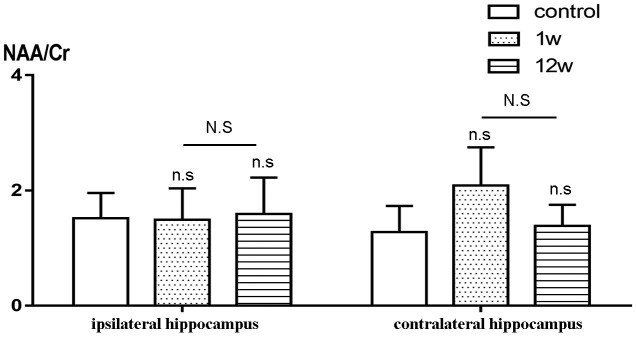
**Comparison of NAA/Cr between ICH group and control group.** n.s P>0.05 compared with control group; N.S P>0.05 compared with 1st w.

The NAA/Cr ratios in the ipsilateral and contralateral hippocampus at weeks 1 and 12 did not significantly differ between the ICH and control groups. Similarly, no difference was found between weeks 1 and 12 among the ICH patients (Figure 6). In addition, no significant correlation was detected between NAA/Cr ratios and cognitive function (MoCA and MMSE) at the selected time points in this study.

## DISCUSSION

The present study demonstrated that deep supratentorial hemorrhage may induce secondary injury to distant regions, which can be detected by DTI early during week 1 after the onset of symptoms. The FA values for basal ganglia nuclei were improved by standard medical treatment, but in the ipsilateral hippocampus, the FA value decreased continuously during the 12 weeks after ICH. Patients with ICH in this study presented a progressive cognitive decline during the study period, and the severity correlated with the FA value for the ipsilateral hippocampus. ROI analysis showed a decrease in FA values for the ipsilateral cerebral peduncle during week 1, which was increased by week 12 and correlated positively with FMS.

In 1850, the British researcher Augustus Waller was the first to describe distal nerve axon and myelin destruction after localized lesions. Subsequent clinical and animal studies revealed distant damage and impaired neurological function secondary to focal cerebral lesions, now referred to as Wallerian degeneration (WD) [18–21]. WD is associated with a decline in diffusion capacity [8, 22, 23], which can be detected using DTI within days or weeks after cerebral injury [21, 24, 25]. In the present study, we confirmed distal regional injuries as early as the 1st week after deep supratentorial hemorrhage. Brain areas with decreased FA values were found not only in motor-related areas, but also in cognition-related areas, which was consistent with patients’ clinical manifestations. A significant correlation was found between ipsilateral cerebral peduncle FA at week 1 and FMS at week 12. We therefore hypothesized that performing DTI during week 1 after the onset of ICH could help to predict motor function at 3 months.

In addition to impaired motor function, a decline of cognition is a common and serious outcome after ICH. This cognitive dysfunction after ICH not only adversely affects patients’ life quality, it also hinders the recovery of neurological function [26]. Fortunately, one study found that vascular cognitive impairment is reversible when treated at an early stage [27]. In those cases, therefore, early detection and intervention are crucial to improving the prognosis. Although the hippocampus is the most critical region for cognition and memory [15], acute ischemic stroke elsewhere in the brain may also cause cognitive dysfunction [28]. For instance, Yakushiji et al revealed an association between microbleeds in the basal ganglia and cognitive impairment [29]. In addition, Kokubo et al found a decline in executive function in patients who hemorrhaged within the putamen [30]. Other studies [31, 32] confirmed that cortical infarction can lead to hippocampal atrophy, metabolic dysfunction, and memory decline. Recently, Shi et al detected significant cognitive impairment after basal ganglia hemorrhage, accompanied by reduced spine density on ipsilateral hippocampus neurons [33]. In the present study, we found that cognitive function is diminished as early as week 1 after ICH, and whole brain-based analysis of DTI data showed a decline in FA values in the ipsilateral hippocampus. By week 12 after ICH, cognitive function had deteriorated, and there was a further decline in the FA value in the ipsilateral hippocampus. The mechanism by which distant regions are injured after a local cerebral hemorrhage has not been fully elucidated, but it may be related to axonal degeneration, neurotransmitter dysregulation, inflammatory responses, neurotoxic factors and/or activated autophagy. Notably, the FA value for the contralateral hippocampus was significantly higher at week 12 than week 1, suggesting the contralateral hippocampus may play a compensatory role after the secondary injury to the ipsilateral hippocampus. In addition, we found that the ipsilateral hippocampus FA values during weeks 1 and 12 after ICH correlated with the MMSE and MoCA, suggesting this FA value may be predictive of cognitive function after ICH.

Our results indicate that DTI is highly sensitive and specific for detecting distant injuries after local ICH, and it can be used for early prediction of functional prognosis. Kantarci et al found that the concentration of metabolic products may change prior to structural changes [34]. NAA is produced in the mitochondria of nerve cells; it is usually found in living cells and axons, and is recognized to be a marker of neuronal activity. Cr is stable in the brain and is often used as an internal reference to calculate metabolic product ratios in order to eliminate differences among individuals [35]. In this study, we used the NAA/Cr ratio as a biomarker of hippocampal metabolites. Despite studies demonstrating secondary hippocampal injury and cognition impairment after ischemic stroke using the 1H-MRS technique [31, 36], there was as yet no evidence of hippocampal metabolic disturbance secondary to ICH due to the lack of study of 1H-MRS in ICH. In the present study, no significant metabolic abnormality after ICH was detected, which is different from secondary changes seen after ischemic stroke. There are several possible reasons for this discrepancy. First, in contrast to ischemic stroke, the pathogenesis of secondary injury to distal regions after cerebral hemorrhage may not due to metabolic disorders. Second, paramagnetic material after ICH may interfere with 1H-MRS, leading to erroneous results. Third, the skull and cerebrospinal fluid may interfere with the 1H-MRS scan. Lastly, the small sample size may have contributed to the lack of a statistically significant difference.

No significant differences for MD values were found between the ICH and control groups, nor was there a within-group difference between ICH group at week 1 and at week 12. The MD values reflect the overall diffusion ability and do not depend on the direction of diffusion. Although the dissolution of the myelin sheath and axon can lead to increases in water molecule motion and MD values, the proliferation of glial cells during WD restricts the movement of water molecules, which we assume is the reason for the lack of obvious changes in MD in our study.

There were several limitations to our study. First, the patients were recruited after an ICH attack, so we could not ensure that some patients did not experience mild cognitive decline before the onset of ICH. To reduce this error, we carefully investigated the patients' daily performance prior to admission and excluded patients with MCI. Second, the sample size is very small, which makes it more susceptible to selection bias. Third, the follow-up period was 12 weeks, which was not enough to determine a long term prognosis. A longer period of follow-up is therefore necessary.

In conclusion, patients with deep supratentorial ICH suffer from widespread brain damage, including to the ipsilateral temporal lobe, frontal lobe and hippocampus. Secondary injury to the ipsilateral cerebral peduncle was detected at week 1 and 12. Injury to the ipsilateral hippocampus persisted for 12 weeks and combined with the continued decline of cognition. FA values for the ipsilateral cerebral peduncle and ipsilateral hippocampus 1 week after the onset of ICH may be predictive of motor and cognition prognosis, respectively.

## MATERIALS AND METHODS

### Patient enrolment

**Figure 7:**
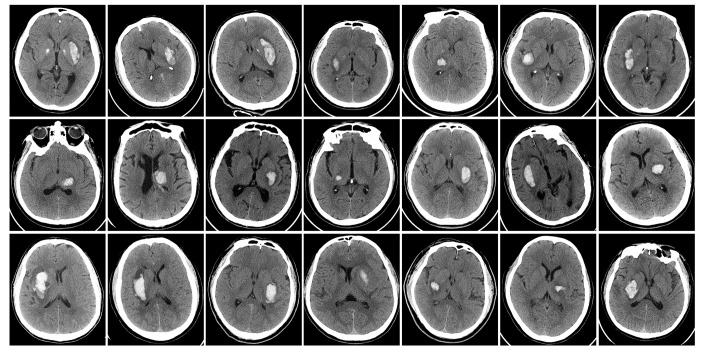
**Baseline head CT of ICH patients immediately after admitting hospital.**

The Ethics Committee of the Clinical Medical College of Yangzhou University approved this study, and written informed consent was obtained from each subject. ICH patients hospitalized in the Department of Neurology and undergoing medical therapy were consecutively enrolled between June 2016 and December 2016. Inclusion and exclusion criteria are listed in Table 3. Single deep supratentorial intracerebral hemorrhage, including in the basal ganglia and thalamus, was diagnosed by CT (Figure 7). A total of 21 ICH patients were included (among which 16 were male); the mean age was 59.29 ± 9.89 years (range from 45-75). Ten healthy volunteers matched in age, sex and education level were recruited as a control group.

**Table 3:** Inclusion and exclusion criteria.

**Inclusion criteria**
1) deep supratentorial intracerebral hemorrhage diagnosed by CT (see Figure 1)
2) admission to hospital within 24 hours from symptom onset
3) age between 18 to 80
5) hemorrhage volume less than 30 ml calculated using the Coniglobus formula
6) clear consciousness and stable vital signs enabling effective MRI examination and neurological scale assessment.
**Exclusion criteria:**
1) hematoma ruptured into ventricles or subarachnoid space
2) secondary ICH due to aneurysm, arteriovenous malformation or other cause
3) history of stroke or brain trauma
4) history of dementia, depression or Parkinson's disease, which could lead to cognitive decline
5) white matter lesion graded 3 according to DSWMH (deep or subcortical white matter hyperintensity) and PVH (periventricular hyperintensity) [37]
6) accompanied by severe infection, liver failure, kidney failure, abnormal coagulation mechanism, thrombocytopenia, tumor and other disease
7) contraindications of MRI, such as cardiac pacemaker or artery stent implantation and claustrophobia.

### Imaging protocols

MRI was performed on a 3.0 Tesla scanner with an 8 channel head coil (Discovery MR750, GE Healthcare, USA). ICH patients underwent two MRI scans, during the 1st week ± 2 days and the 12th week ±2 days after the onset of ICH. The healthy controls underwent MRI scanning just once. MRI sequences included T1WI, T2WI, FLAIR, DWI, DTI and 1H-MRS. The DTI data were acquired using single-shot spin-echo echo-planar imaging (SS-SE-EPI): 35 non-collinear direction; b values, 0 s/mm^2^ and 1000 s/mm^2^; TR, 8000 ms; TE, 80.8 ms; FOV, 256 × 256 mm^2^; number of excitations (NEX), 1; slice thickness, 2 mm with no gap. 3D chemical shift imaging technology (3D-CSI) was applied to acquire 1H-MRS data on the basis of T1WI: TR, 1000 ms; TE, 44 ms; average number of acquisitions (Average), 1; water suppression rate, over 97%; and half bandwidth (FWHM), < 15 Hz. The region of interest of MRS was prescribed on the bilateral hippocampus.

### Clinical assessment

Clinical data were collected prospectively from patients and controls. Neurological function was assessed 2 h before MRI performance. The National Institutes of Health Stroke Scale (NIHSS) and FMS were used to evaluate ICH severity, with a higher NIHSS and lower FMS indicating more serious impairment of motor function. MMSE and MoCA were used to evaluate cognitive function, with a lower score indicating a more serious cognitive impairment. An overall good outcome was defined as modified Rankin Scale (mRS) ≤2 during the 12th week after ICH onset.

### Imaging analysis

DTI data preprocessing was carried out using FSL 5.0 (FMRIB Software Library, http://www.fmrib.ox.ac.uk/fsl/) based on Oracle VM VirtualBox. First, the lesion located in the left brain was flipped to the right along the mid-sagittal line and the b0 image of each subject was skull-stripped using the brain extraction tool. The data were then corrected for head motion and image distortion caused by eddy currents, and the diffusion sensitizing gradients (“bvecs”) were rotated to correct for motion. Using FDT, the diffusion tensor model was fit to the data, from which FA and MD images were calculated. For DTI data analysis, SPM8 (Statistical Parametric Mapping, SPM8-http://www.fil.ion.ucl.ac.uk/spm/) based on MATLAB was used. The diffusion maps for each subject were normalized to the Montreal Neurologic Institute (MNI) space using the echo plane image (EPI) template. Thereafter, we carried out a two-sample t-test in SPM8. Brain areas showing significant differences between the ICH and control groups were overlaid on the standard CH2 structure image template, showing the cross section cutting layer. The statistical threshold set as P < 0.001, and voxel clusters over 100 voxels were displayed.

Templates for the bilateral hippocampus mask were made from the automatic anatomical labeling (AAL) using the WFU PickAtlas (https://www.nitrc.org/projects/wfu_pickatlas/). The bilateral cerebral peduncle mask was extracted from the JHU ICBM-DTI-81 white-matter atlas of FSL, after which the size was resampled to make it consistent with diffusion parameter after standardization. The masks were applied to the FA and MD map after smoothing, and FA and MD values were calculated for each patient's hippocampus and cerebral peduncle.

To process 1H-MRS data, we transmitted and analyzed the scanned data using GE ADW4.6 FUNCTIONAL software. Inhibition of the residual water, performance of the Fourier transform, and adjustment of the baseline and phase were completed automatically to obtain peak values for NAA, Cr, and the NAA/Cr ratio.

### Statistical analysis

Clinical and imaging data were analyzed using SPSS19.0. Normally distributed continuous variables are presented as the mean ± standard deviation (SD), and unpaired t-tests were applied to analyze differences between the ICH and control groups; paired t-tests were used to analyze within-group differences across time. Categorical variables are presented as numbers (percentage), and the Chi-square or Fisher’s exact test was used for analysis. The correlations between FA values, NAA/Cr and MMSE, MoCA and FMS were analyzed using Spearman analysis. Statistical significance was defined as *P*< 0.05.

## Supplementary Material

Supplementary Figure 1

Supplementary Tables
