# Supplementary material for: Secondary injury to distal regions after intracerebral hemorrhage influence neurological functional outcome
Source: Aging (Albany NY). 2020 Mar 8;12(5):4283–98. doi: 10.18632/aging.102880 (PMC7093199; doi:10.18632/aging.102880)
Supplement: Supplementary Figure 1 [file aging-12-102880-s001..pdf]

SUPPLEMENTARY FIGURE

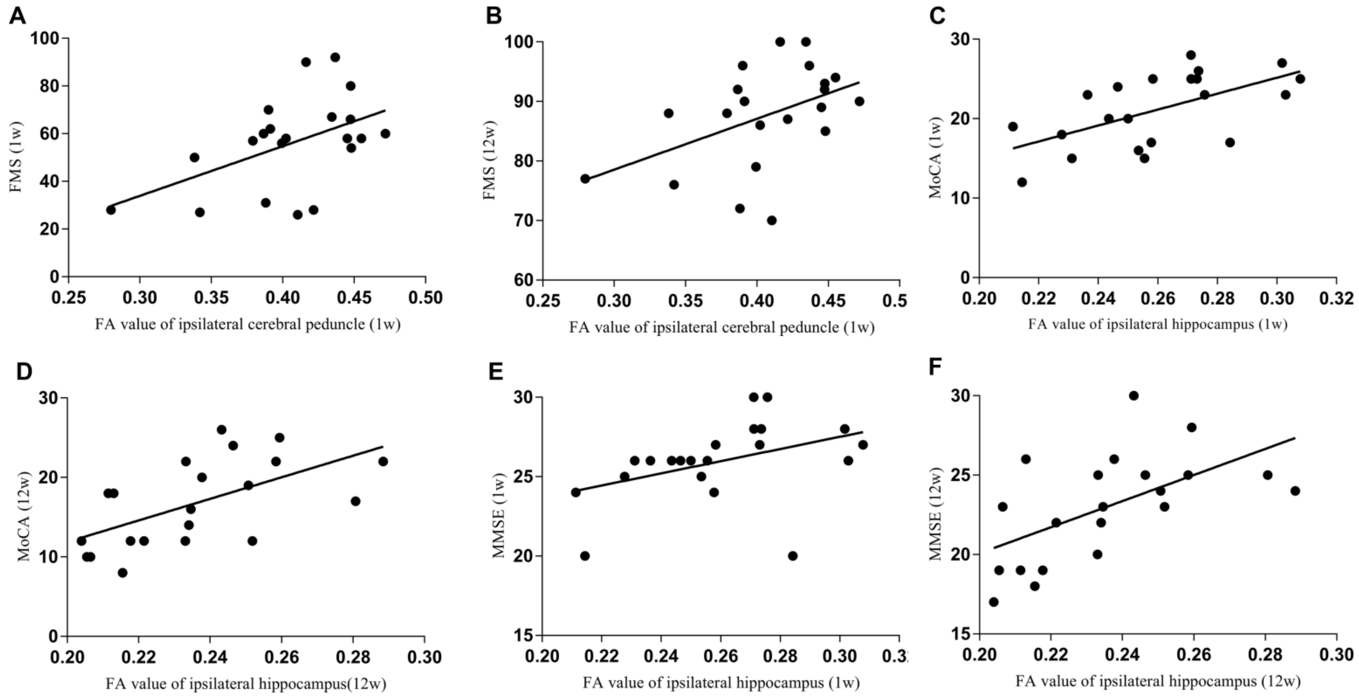

Supplementary Figure 1. Association between FA value and neurological functional outcome. (**A**) Association of patients' ipsilateral cerebral peduncle FA value at 1st w was associated with FMS at 1st w. (**B**) Association of patients' ipsilateral cerebral peduncle FA value at 1st w was associated with FMS at 12th w. (**C**) Association of patients' ipsilateral hippocampus FA value at 1st w was associated with MoCA at 1st w. (**D**) Association of patients' ipsilateral hippocampus FA value at 12th w was associated with MoCA at 12th w. (**E**) Association of patients' ipsilateral hippocampus FA value at 1st w was associated with MMSE at 1st w. (**F**) Association of patients' ipsilateral hippocampus FA value at 12th w was associated with MMSE 12th w.
