# Supplementary material for: Secondary injury to distal regions after intracerebral hemorrhage influence neurological functional outcome
Source: Aging (Albany NY). 2020 Mar 8;12(5):4283–98. doi: 10.18632/aging.102880 (PMC7093199; doi:10.18632/aging.102880)
Supplement: Supplementary Tables [file aging-12-102880-s002..pdf]

SUPPLEMENTARY TABLES

Supplementary Table 1. Brain areas of decreased FA value among ICH patients compared to healthy control at 1st week.

	Structure	Voxels	Peak intensity	Peak MNI coordinate		
				X	Y	Z
Cluster1	Total	2534	8.5932	24	-24	22
	Right Cerebrum	2516				
	White Matter	2005				
	Gray Matter	457				
	Temporal Lobe	456				
	Frontal Lobe	298				
	Superior Temporal Gyrus	215				
	Lentiform Nucleus	213				
	Insula	209				
	Temporal_Sup_R (aal)	194				
	Putamen_R (aal)	187				
	brodmann area 13	93				
	brodmann area 41	90				
	Caudate_R (aal)	90				
	Hippocampus_R (aal)	83				
	Parietal Lobe	82				
	Thalamus_R (aal)	69				
	Transverse Temporal Gyrus	48				
	Pallidum_R (aal)	25				
	Inferior Parietal Lobule	25				
	Medial Globus Pallidus	24				
	Midbrain	24				
	Claustrum	23				
	Rolandic_Oper_R (aal)	22				
	Right Brainstem	18				
	Amygdala_R (aal)	15				
	Precuneus_R (aal)	15				
Cluster2	Total	418	5.4448	-20	18	4
	Left Cerebrum	418				
	White Matter	338				
	Gray Matter	80				
	Putamen_L (aal)	68				
	Lentiform Nucleus	67				
	Sub-Gyral	36				
	Frontal Lobe	36				
	Caudate_L (aal)	31				
	Thalamus_L (aal)	26				
	Thalamus	13				

Supplementary Table 2. Brain areas of decreased FA value among ICH patients compared to healthy control at 12 week.

	Structure	Voxels	Peak intensity	Peak MNI coordinate		
				X	Y	Z
Cluster1	Total	154	4.3403	24	20	-8
	Right Cerebrum	154				
	White Matte	132				
	Putamen_R (aal)	115				
	Gray Matter	22				
	Claustrum	19				
	Frontal Lobe	8				
	Sub-Gyral	7				
	Lentiform Nucleus	2				
Cluster2	TOTAL	313	4.527	32	-18	10
	Right Cerebrum	313				
	White Matter	196				
	Gray Matter	111				
	Lentiform Nucleus	83				
	Insula_R (aal)	29				
	Claustrum	26				
	Hippocampus_R (aal)	17				
	Lateral Globus Pallidus	15				
	Temporal Lobe	12				
	Sub-Gyral	11				
	Thalamus_R (aal)	10				
	Midbrain	2				

Supplementary Table 3. Brain areas of increased FA value at 12th week compared with 1st week among ICH patients.

	Structure	Voxels	Peak intensity	Peak MNI coordinate		
				X	Y	Z
Cluster1	Total	429	3.6641	-36	-46	8
	Left Cerebrum	429				
	White Matter	339				
	Sub-Gyral	291				
	Temporal Lobe	277				
	Parahippocampa Gyrus	34				
	Temporal_Inf_L (aal)	33				
	Hippocampus_L (aal)	31				
	Gray Matter	25				
	Occipital Lobe	20				
Cluster2	TOTAL	315	4.2843	20	20	4
	Right Cerebrum	315				
	White Matter	293				
	Frontal Lobe	121				
	Sub-Gyral	111				
	Caudate_R (aal)	101				
	Putamen_R (aal)	45				
	Gray Matter	21				
Cluster3	TOTAL	416	4.1084	24	-32	24
	Right Cerebrum	416				
	White Matter	364				
	Sub-lobar	213				
	Extra-Nuclear	161				
	Sub-Gyral	156				
	Temporal Lobe	143				
	Gray Matter	49				
	Insula	49				
	Parietal Lobe	28				
	Hippocampus_R (aal)	6				
Cluster4	TOTAL	257	3.8768	22	-16	0
	Right Cerebrum	255				
	Lentiform Nucleus	179				
	Gray Matter	179				
	Lateral Globus Pallidus	93				
	White Matter	76				
	Thalamus_R (aal)	41				
	Putamen_R (aal)	11				
	Midbrain	5				
	Right Brainstem	2				
Cluster5	TOTAL	165	3.7888	26	-56	-6
	Right Cerebrum	163				
	White Matter	135				
	Occipital Lobe	132				
	Lingual_R (aal)	91				
	Sub-Gyral	68				
	Fusiform_R (aal)	29				

	Gray Matter	27				
	brodmann area 19	26				
	Temporal Lobe	19				
Cluster6	TOTAL	125	3.7124	38	-4	20
	Right Cerebrum	125				
	White Matter	84				
	Insula	74				
	Gray Matter	38				

Supplementary Table 4. Brain areas of decreased FA value at 12th week compared with 1st week among ICH patients.

	Structure	Voxels	Peak intensity	Peak MNI coordinate		
				X	Y	Z
Cluster1	TOTAL	115	-4.5354	22	-34	0
	Right Cerebrum	107				
	Hippocampus_R (aal)	68				
	White Matter	54				
	Gray Matter	45				
	Parahippocampa Gyrus	29				
	Thalamus	29				
	Temporal Lobe	15				
Cluster2	TOTAL	129	-3. 7471	16	-58	12
	Right Cerebrum	127				
	Limbic Lobe	115				
	Posterior Cingulate	108				
	White Matter	95				
	Precuneus_R (aal)	57				
	Lingual_R (aal)	36				
	Calcarine_R (aal)	34				
	Gray Matter	28				
	brodmann area 29	14				
